# Comparison of Lymphocyte Populations in Patients With Dobrava or Puumala orthohantavirus Infection

**DOI:** 10.3389/fcimb.2020.566149

**Published:** 2020-10-16

**Authors:** Katarina Resman Rus, Andreja Nataša Kopitar, Miša Korva, Alojz Ihan, Miroslav Petrovec, Tatjana Avšič-Županc

**Affiliations:** Faculty of Medicine, Institute of Microbiology and Immunology, University of Ljubljana, Ljubljana, Slovenia

**Keywords:** orthohantavirus, Puumala virus, Dobrava virus, hemorrhagic fever with renal syndrome, flow cytometry, PBMCs, T lymphocytes, NK cells

## Abstract

Hemorrhagic fever with renal syndrome (HFRS), caused by Dobrava (DOBV) and Puumala (PUUV) orthohantaviruses, is an endemic disease in Slovenia. DOBV is mainly responsible for a more severe disease, whereas PUUV usually causes a milder form. Therefore, the aim of our study was to determine whether any differences in lymphocyte population in patients infected with these two viruses exist. Mononuclear cells from peripheral blood (PBMCs) were isolated from DOBV or PUUV infected patients and different lymphocyte subpopulations were analyzed with flow cytometry. Decreased concentrations of lymphocyte subpopulation were observed in DOBV and in PUUV infected patients compared with a healthy control, which was especially evident in DOBV infected patients. The lower values of T cells are likely due to the extravasation of the activated cells from the circulation to the infected tissue. Higher percentage of NK cells were detected in DOBV infected patients in comparison to PUUV infected patients, which could be associated with a more severe HFRS caused by DOBV. PUUV infected patients had a significantly higher concentration of activated T cell subsets, expressing markers CD25, CD69, and HLA-DR in comparison to DOBV infected patients. Higher activation of T cell subsets in PUUV infected patients could be a contributor to a milder HFRS. Further studies are necessary to elucidate the relation between the protective and the harmful role of activated lymphocytes subsets in HFRS pathogenesis.

## Introduction

Orthohantaviruses cause two typical syndromes: hemorrhagic fever with renal syndrome (HFRS) in Europe and Asia, and hantavirus cardiopulmonary syndrome (HCPS) in the Americas. In Slovenia HFRS is caused by the Dobrava virus (DOBV) and the Puumala virus (PUUV). DOBV is mainly responsible for a more severe disease, whereas PUUV usually causes a milder form. However, the clinical severity of HFRS varies greatly and in Slovenia both a severe and a mild clinical course of the disease have been observed, with an overall case fatality rate of 4.5% (Avsic-Zupanc et al., 1999; Pal et al., 2005).

Endothelial cells are the main target of hantavirus infection, which is not cytopathogenic (Mackow and Gavrilovskaya, 2009). The hallmark of HFRS is vascular permeability, which is probably caused by an excessive innate immune response, especially by pro-inflammatory cytokines, that affect barrier integrity (Terajima and Ennis, 2011).

Cytotoxic lymphocytes including NK and CD8^+^ T cells have an important role in HFRS pathogenesis (Van Epps et al., 2002; Bjorkstrom et al., 2011; Lindgren et al., 2011; Terajima and Ennis, 2011). NK cells are at the frontier between innate and adaptive immunity and can be activated by virus-induced cytokines (Dokun et al., 2001). Robust NK cell activation, especially CD56^dim^ NK cells, and proliferation has been observed in HFRS patients (Bjorkstrom et al., 2011; Braun et al., 2014). However, the role of NK cells in HFRS pathogenesis is not fully understood, since it was shown that endothelial cells infected with a hantavirus are protected against the NK cell-mediated killing (Gupta et al., 2013).

T cell activation occurs in the acute phase of HFRS (Huang et al., 1994; Markotic et al., 1999). Hantavirus infection induces differentiation of dendritic cells, and subsequently antigen presenting cell (APC) transition and T cell stimulation, since induction of memory T cells with long lasting protection is found in patients with the Andes virus infection (Manigold et al., 2010). A strong response of CD8^+^ cytotoxic lymphocytes and a lesser extension of CD4^+^ T cells contribute to HFRS pathogenesis (Rasmuson et al., 2011). CD8^+^ and CD4^+^ T cells may modulate effector responses through different inhibitory receptors during an acute hantaviral infection (Lindgren et al., 2011). However, the capillary leakage may be mediated by cytokines produced by T cells, such as tumor necrosis factor (TNF)-α, interleukin (IL)-2, IL-6, and interferon (IFN)-γ (Linderholm et al., 1996; Sadeghi et al., 2011; Saksida et al., 2011; Terajima and Ennis, 2011; Khaiboullina et al., 2014; Baigildina et al., 2015; Morzunov et al., 2015). Regulatory CD4^+^ T cells (T_reg_) suppress proinflammatory and effector T cells activity and have an important role in the persistent hantavirus infection in rodents (Belkaid and Rouse, 2005; Li and Klein, 2012). However, T_reg_ cell response correlates with the severity of PUUV-induced HFRS, which indicate that the suppressive function of T_reg_ cells is deleterious (Koivula et al., 2014).

Although different immune studies have been conducted, immunophenotypic changes of lymphocytic population in patients with HFRS are not fully characterized. Moreover, some inconsistencies are observed in the analysis of lymphocytic populations, depending on which orthohantavirus was examined. The aim of our study was to investigate the differences in PBMC profiles of patients infected with DOBV or PUUV and to determine whether the observed differences could be associated with different clinical course of the disease.

## Materials and Methods

### Ethics Statement

The study was approved by the Republic of Slovenia National Medical Ethics Committee (69/03/12 and 30/04/15). All participants gave an oral and a written informed consent. The study was conducted according to the principles expressed in the Declaration of Helsinki.

### HFRS Patients

A total of 36 patients (29 males and 7 females) with confirmed HFRS, hospitalized in different Slovenian hospitals between the years 2000 and 2015, were involved in the study. As a control group, 26 healthy adult volunteers (10 males and 16 females) were included. Clinical diagnosis of all patients was laboratory confirmed with serological and molecular tests as described previously (Avsic-Zupanc et al., 1999; Saksida et al., 2008; Korva et al., 2009). Viral RNA load was measured in patients' plasma samples using Quantitative One-Step real-time reverse transcriptase polymerase chain reaction (qRT-PCR) as described previously (Korva et al., 2013). Briefly, viral RNA was isolated from patients' plasma samples using EZ1 Virus Mini Kit v2.0 according to manufacturer's instructions (Qiagen). For qRT-PCR reaction TaqMan Fast Virus 1-Step Master Mix was used according to manufacturer's instructions (Applied Biosystems). For DOBV detection primers DOB-S F1 (TTGTTCCTGTTTGCTGGAAAATGAT), DOB-S R1 (CGGGTTGAAGAATGGCTTGAC) and probe DOB-S-MGB-P (FAM-CCGTGCAAGCTAC) were used. For detection of virus PUUV primers PUU D (GGAGTAAGCTCTTCTGC), PUU L (ACATCATTTGAGGACAT) and probe PUU-MGB (VICAGACCAAAGCATTTATATG) were used. qRT-PCR reaction was performed on ABI 7500 Fast instrument (Applied Biosystems) under conditions of 5 min at 50°C, 20 s at 95°C and 45 cycles of 3 s at 95°C, 30 s at 55°C and 30 s at 60°C. DOBV and PUUV RNA was quantified using calibrated synthetic standard gBlock (IDT). For each patient a detailed medical chart was collected and a differential white blood cell count was analyzed.

### Isolation of PBMCs

PBMCs were isolated from EDTA blood samples and were purified using Ficoll-Paque™ PLUS (GE Healthcare, Uppsala, Sweden) density gradient centrifugation on Leucosep™ tubes (Greiner Bio-One, GmbH, Germany) according to the manufacturer's instructions. Isolated PBMCs were re-suspended in RPMI 1640 (Sigma, UK) with 80% fetal bovine serum (EuroClone, Pero, Italy) and 10% dimethyl sulfoxide and cryopreserved until further analysis.

### Initial *in vitro* Experiment of PBMCs Stimulation

PBMCs from a healthy donor were thawed and re-suspended in RPMI 1640 with 10% inactivated human serum (Sigma, UK), 1% L-glutamine (Gibco, UK), and 2% antibiotics (penicillin 100 U/mL, streptomycin 0.1 mg/mL). The final concentration of PBMCs was 3 × 10^6^ cells/mL in a single well of a 24-well plate (Costar, Buckinghamshire, UK). Hantaviruses DOBV and PUUV were grown on a VERO E6 cell line and were tested to be *Mycoplasma* free (ATCC commercial kit). Viruses were cleaned through NAP-5 columns prepacked with Sephadex G-25 DNA Grade resin (Illustra, GE Healthcare, USA) and re-suspended in saline. PBMCs were stimulated with DOBV or PUUV at multiplicity of infection 0.1 and incubated at 37°C with 5% CO_2_ for 48 h. As negative control non-stimulated PBMCs were analyzed.

### Fluorescence Labeling and Flow Cytometry Analysis of *in vitro* Stimulated PBMCs

*In vitro* stimulated PBMCs were centrifuged at 1,600 rpm for 5 min and re-suspended in phosphate-buffered saline (PBS). PBMCs were incubated in the dark for 20 min with the monoclonal antibodies (MAbs) mixture for detection of different PBMCs subtypes. Combination of MAbs conjugated with fluorochromes against the following cell surface structures was used: CD3-FITC, CD16+CD56-PE, CD8-PEcy7, CD14-PerCP-Cy5.5, CD4-APCcy7, CD19-V450, and CD45-V500 (Becton Dickinson Bioscience, Mountain View, SA, USA). Labeled PBMCs were washed with PBS and then BD Cytofix™ Fixation Buffer (Becton Dickinson) was added for subsequent immunofluorescent intracellular staining and Hantavirus nucleoprotein detection. Monoclonal Hantavirus NP Antibody (Thermo Fisher Scientific, USA) labeled with Alexa Fluor® 647 (APEX^TM^ Antibody Labeling Kits, Invitrogen, UK) was added in a concentration of 0.005 μg/μl. Fixed PBMCs were centrifuged 5 min at 1,600 rpm and washed twice with PBS. Labeled PBMCs were re-suspended in 200 μl PBS. Data were acquired on BD FACSCanto II using BD FACSDiva 8.0.1 (Becton Dickinson) and analysis was performed using BD Diva 6.1.

### Antibodies for Flow Cytometry Analysis of PBMCs From HFRS Patients

Four different combinations of commercially available monoclonal antibodies (MAbs) conjugated with fluorochromes against the following cell surface structures were used: (1) commercial mixture of MAbs Lymphogram® (CD3-PE, CD19-FITC, CD4-PerCP, CD8-FITC, CD56-PE) (Cytognos, Salamanca, Spain), (2) CD25-FITC, CD127-PE, CD4-PerCP-Cy5.5, (3) CD69-FITC, CD16-PE, CD3-PerCP-Cy5.5, CD56-APC and (4) HLA-DR-PerCP-cy5, CD69-FITC, CD3-APC, CD4-APC-Cy7, CD8-V450. All MAbs except Lymphogram® were purchased from Becton Dickinson Bioscience (Mountain View, SA, USA).

### Fluorescence Labeling and Flow Cytometry Analysis of PBMCs From HFRS Patients

**Figure 1:**
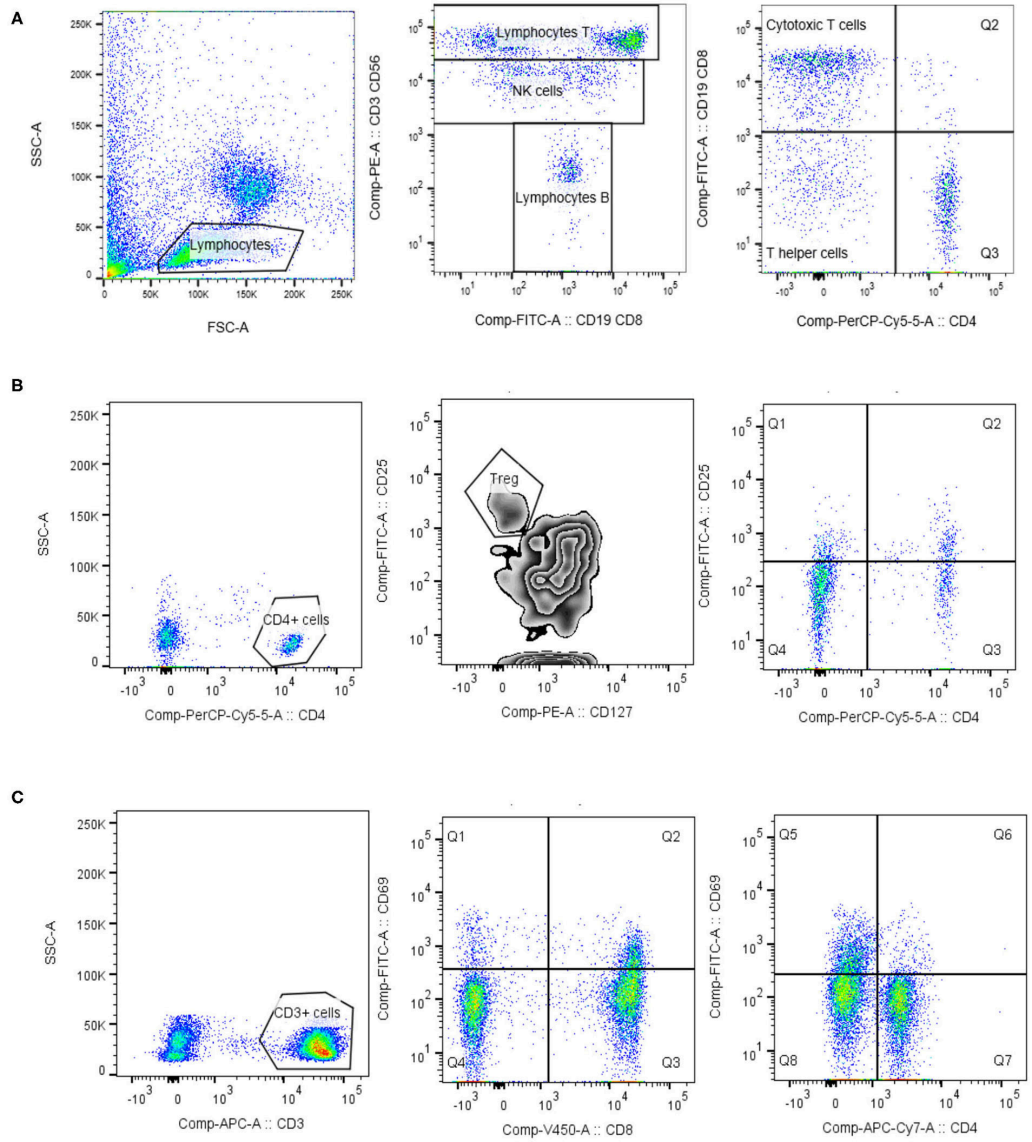
Lymphocytes phenotypisation. Lymphocytes were identified based on the forward/side scatter properties. For the identification of lymphocyte T, lymphocyte B and NK cells the commercial mixture of monoclonal antibody Lymphogram (Cytognos) were used **(A)**. For the determination of regulatory T cells (Treg) and activated helper T cells, expressing IL-2 receptor (CD25), lymphocytes were further gated on CD4 positive cells. Treg cells expressed high level of IL-2 receptor (CD25) and low/negative level of IL-7α receptor (CD127) **(B)**. Activated T cells (CD3, CD4, and CD8) were identified as HLA-DR+ cells, or CD69+ cells **(C)**. Data analysis was performed using BD Diva 6.1 and FlowJo version 10.1 (TreeStar).

Isolated PBMCs were thawed, re-suspended in RPMI 1640 and centrifuged at 1,600 rpm for 5 min. PBMCs were re-suspended in phosphate-buffered saline (PBS) at concentration of 10^6^ cells/ml and incubated with the appropriate MAbs mixture in the dark for 20 min, then lysis buffer (Becton Dickinson) was added and incubated in the dark for 10 min. Fixed PBMCs were centrifuged 5 min at 1,600 rpm and washed twice with PBS. Labeled PBMCs were re-suspended in 200 μl PBS. Normal values were performed on 26 healthy adult volunteers. Data were acquired on BD FACSCanto II using BD FACSDiva 8.0.1 (Becton Dickinson). The gating strategies of the flow cytometry analysis are shown in Figure 1. Data analysis was performed using BD Diva 6.1 and FlowJo version 10.1 (TreeStar).

### Soluble IL-2 Receptor Immunoassay

Concentration of soluble IL-2 receptor was measured in acute serum samples of 31 HFRS patients. Chemiluminiscent IL-2R assay was used according to manufacturer instructions (Siemens). Briefly, a bead coated with murine monoclonal anti-IL-2R antibody were mixed with alkalinephosphatase conjugated to a rabbit anti-IL-2R polyclonal antibody and sample. Solid phase, reagent, and sample are incubated together. Soluble IL-2R in the sample binds to the solid phase antibody and reagent antibody forming a sandwich complex on the bead. Unbound enzyme conjugate is then removed by a centrifugal wash. Finally, chemiluminescent substrate is added to the bead and signal is generated in proportion to the bound enzyme. For analyses, Immulite 2000 XPi Immunoassay System was used (Siemens). Results of measurements were reported as units U/mL.

### Statistical Analysis

GraphPad Prism version 7 for Windows (GraphPad Software, San Diego, CA, USA) was used for the analyses. The non-parametric Kruskal–Wallis test, followed by Dunn's post analysis was performed for lymphocyte populations' analysis. The non-parametric Mann-Whitney test was performed for a viral RNA loads comparison between DOBV and PUUV infected patients. The non-parametric Spearman correlation test with 95% confidence interval was performed to compute correlations. All statistical tests were two-tailed and a *p*-value below 0.05 was considered significant.

## Results

### Patients and Clinical Data

PBMCs were isolated from EDTA blood samples of 36 HFRS patients during the acute phase of infection, in the first week of hospitalization (median on the 3^th^ day of hospitalization and on the 7^th^ self-reported day of disease). The median age of included patients was 37 years (min 18, max 70). Among HFRS patients, 15 patients were infected with DOBV (14 males and 1 female) and 21 with PUUV (15 males and 6 females). The median age of DOBV infected patients was 36 years (min 18, max 70) and blood samples were collected in median on the 3.5 day of hospitalization (median on the 8^th^ self-reported day of disease). The median age of PUUV infected patients was 37 years (min 22, max 70) and blood samples were collected in median on the 2^nd^- day of hospitalization (median on the 6^th^ self-reported day of disease). The median age of healthy volunteers was 26 years (min 21, max 47). Detailed medical charts were collected for all included patients and representative clinical data about thrombocytes, urea, creatinine, hematocrit, and CRP presented in Table 1 were selected based on our previous study (Pal et al., 2018).

**Table 1:** Representative clinical parameters of HFRS patients.

	**Hematocrit (1)**		**Thrombocytes (10**^****9****^**/L)**		**Urea (mmol/L)**		**Creatinine (μmol/L)**		**CRP (mg/L)**	
	**DOBV**	**PUUV**	**DOBV**	**PUUV**	**DOBV**	**PUUV**	**DOBV**	**PUUV**	**DOBV**	**PUUV**
n	13	19	15	21	14	21	15	21	14	19
Mean	0.4	0.4	92.9	91.1	20.2	11.1	413.7	225.1	61.8	87.5
Median	0.4	0.5	57	78	16	10.4	342	157	50.4	59
Minimum	0.3	0.4	23	27	6.4	2.3	77	67	4	30
Maximum	0.5	0.5	510	271	48.4	25.8	936	629	211	270

In addition, convalescent samples were collected from 15 out of 36 HFRS patients. Samples were collected in median 3 years after acute HFRS (min 1, max 7 years). Convalescent samples were collected from 7 DOBV (6 males, median age 36 years) and 8 PUUV infected patients (7 males, median age 39). Convalescent samples were in both groups of patients collected in median 3 years after recovery (min 1 year, max 7 years).

### Virus Detection in *in vitro* Stimulated PBMCs

**Figure 2:**
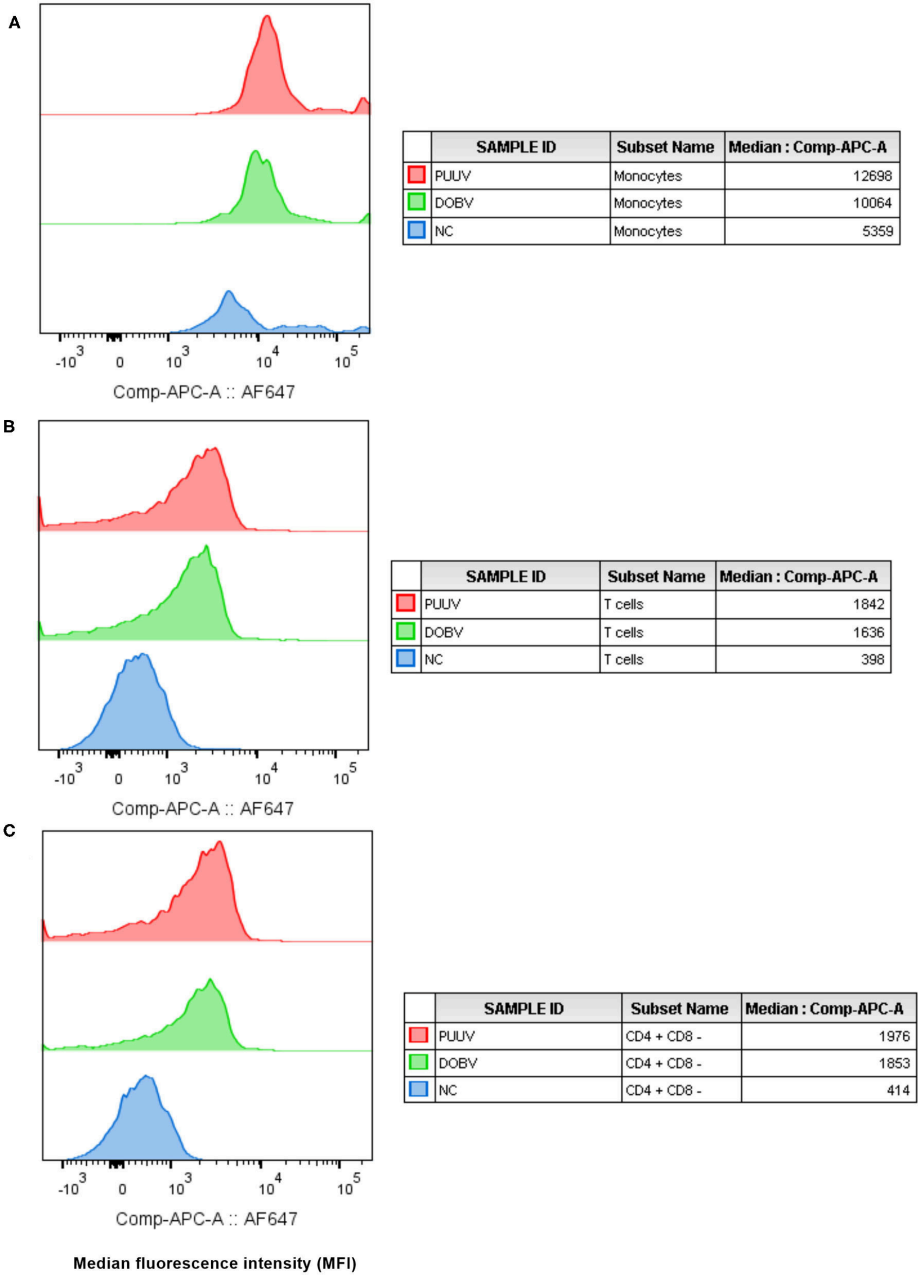
Virus detection in *in vitro* stimulated PBMCs. The mean fluorescence intensity (MFI) of monoclonal antibody anti Hantavirus N protein (labeled with Alexa Fluor 647) was analyzed in monocytes **(A)**, T cells **(B)**, and CD4+ T cells **(C)**, after stimulation with PUUV, DOBV and in no stimulated cells (NC).

To determine which cell populations are infected with hantaviruses, PBMCs were *in vitro* stimulated with DOBV or PUUV and the median fluorescence intensity (MFI) of mAb anti Hantavirus N protein was analyzed with flow cytometry. Results indicated that the Hantavirus N protein was detected in monocytes (*p* = 0.015) (in 31% after DOBV stimulation and in 46.5% after PUUV stimulation) and T cells (*p* = 0.009) (in 1.5% after stimulation with both viruses), especially in CD4+ cells (*p* = 0.009) at 48 h after PBMCs stimulation with DOBV or PUUV (Figure 2). When monocytes were analyzed, fluorescence intensity was 2.1-times higher after stimulation with DOBV and 2.4-times higher after stimulation with PUUV in comparison to the negative control (Figure 2A). When T cells were analyzed, fluorescence intensity was 3.7-times higher after stimulation with DOBV and 4.2-times higher after stimulation with PUUV (Figure 2B) in comparison to the negative control. Moreover, a higher fluorescence intensity of mAb anti Hantavirus N protein was detected when CD4+ cells were analyzed, 3.6-times higher after stimulation with DOBV and 3.8-times higher after stimulation with PUUV compared to the negative control (Figure 2C).

### Lymphocytes in HFRS Patients

**Figure 3:**
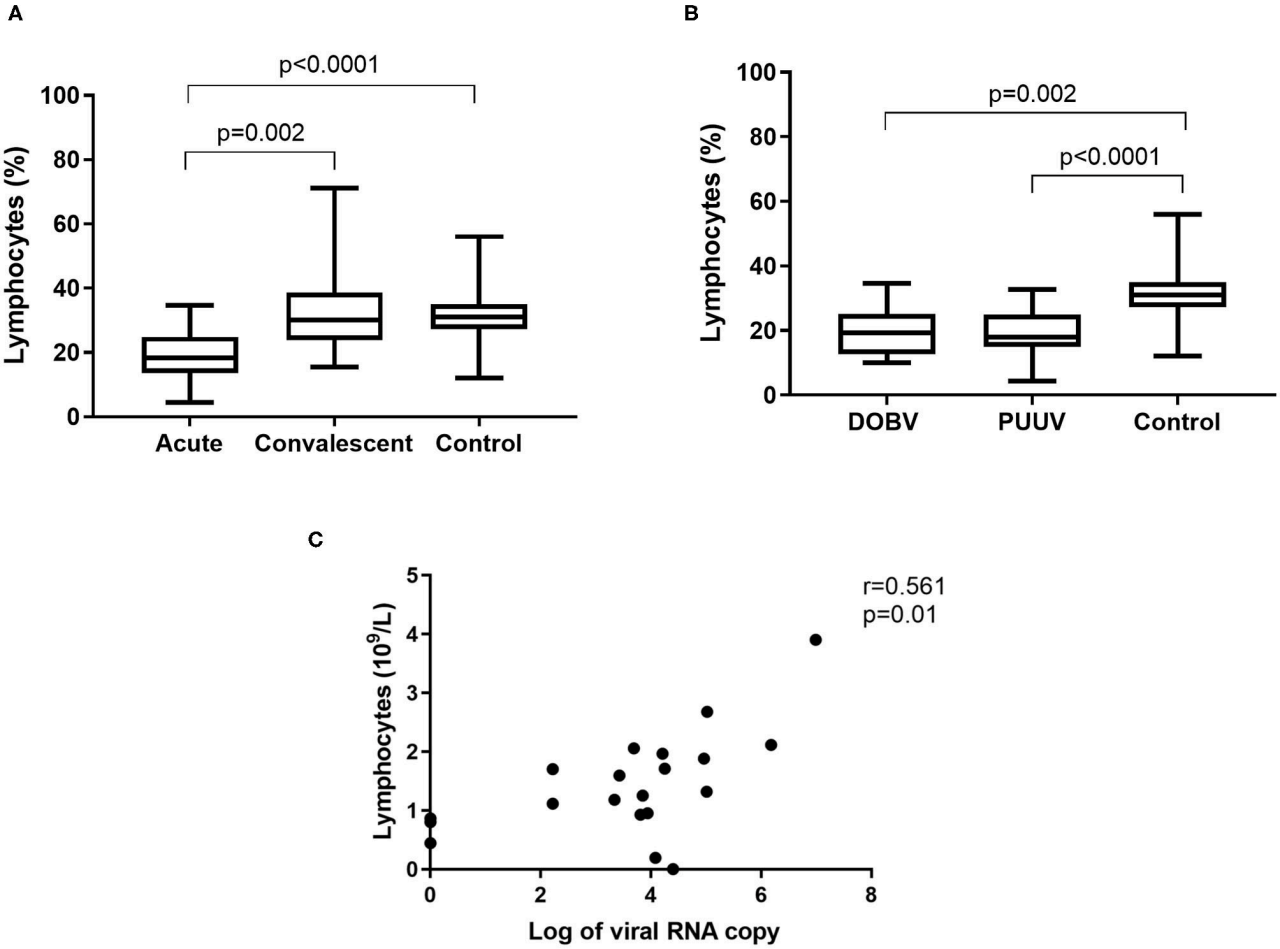
Lymphocytes in HFRS patients. **(A)** Comparison of percentage of lymphocytes between acute and convalescent samples of HFRS patients and healthy control. **(B)** Comparison of percentage of lymphocytes between DOBV and PUUV infected patients and healthy control. **(C)** Correlation between concentration of lymphocytes and viral RNA load in PUUV infected patients (*n* = 20). The non-parametric Kruskal–Wallis test, followed by Dunn's post analysis was used for statistical analyses. The non-parametric Spearman correlation test with 95% confidence interval was performed to compute correlations. A *p*-values below 0.05 were considered statistically significant.

To determine whether differences in lymphocyte numbers exist, the percentage and concentration of lymphocytes between HFRS patients and the healthy control were analyzed. Results indicated that the percentage of lymphocytes was significantly lower in the acute phase of the disease and normalized in the convalescent phase (*p* = 0.002; Figure 3A). Significantly lower percentage of lymphocytes was measured in both groups of patients in comparison to the healthy control (*p* = 0.002 for DOBV, *p* < 0.0001 for PUUV) (Figure 3B). The concentration of lymphocytes was calculated from white blood cell count. Statistically significantly lower concentration of lymphocytes was calculated in HFRS patients (*p* = 0.001 for DOBV, *p* = 0.014 for PUUV) in comparison to the healthy control (Table 2). Moreover, concentration of lymphocytes positively correlates with the viral RNA load in PUUV infected patients (*r* = 0.5612, *p* = 0.01; Figure 3C).

**Table 2:** Different lymphocyte subpopulations analyzed with flow cytometry in DOBV and PUUV infected patients and healthy control.

		**DOBV**	**PUUV**	**Control**	***p*****-values**		
		**n = 15**	**n = 21**	**n =26**	**DOBV vs. PUUV**	**DOBV vs. control**	**PUUV vs. control**
%	Lymphocytes	19 (13–25)	18 (15–25)	31 (27–35)	ns	0.002	< 0.0001
	CD56+	16 (8–22)	10 (7–14)	16 (12–20)	0.043	ns	0.004
	CD3+	66 (57–73)	79 (70−82)	73 (70–77)	0.006	ns	ns
	CD3+CD4+	47 (40–54)	49 (45–58)	44 (38–48)	ns	ns	0.02
	CD3+CD8+	43 (32–49)	40 (33–47)	19 (16–27)	ns	< 0.0001	< 0.0001
	CD4+CD25+	7 (5–9)	9 (6–10)	13 (10–14)	ns	< 0.0001	0.005
	CD4+CD25++CD127^−/low^	7 (6–9)	8 (5–10)	6 (5–7)	ns	ns	0.017
	CD3+CD69+	3 (2–8)	5 (4–7)	4 (2–5)	ns	ns	ns
	CD4+CD69+	4 (3–7)	3 (2–8)	3 (2–5)	ns	ns	ns
	CD8+CD69+	4 (2–8)	5 (4–8)	5 (3–6)	0.036	ns	ns
	CD3+HLA-DR+	15 (9–17)	18 (14–25)	9 (6–10)	ns	0.013	< 0.0001
	CD4+HLA-DR+	10 (4–20)	7 (6–14)	3 (2–4)	ns	0.0001	< 0.0001
	CD8+HLA-DR+	9 (6–14)	15 (11–17)	5 (2–8)	ns	ns	< 0.0001
Concertation (10^9^/L)	Lymphocytes	1.27 (0.79–1.84)	1.65 (1.07–2.09)	2.20 (1.89–2.52)	ns	0.001	0.012
	CD56+	0.16 (0.10–0.42)	0.17 (0.09–0.27)	0.33 (0.25–0.52)	ns	ns	0.001
	CD3+	0.84 (0.46–1.23)	1.08 (0.85–1.59)	1.60 (1.37–1.98)	ns	< 0.0001	0.014
	CD3+CD4+	0.31 (0.26–0.69)	0.59 (0.5–0.94)	0.93 (0.85–1.17)	0.029	< 0.0001	0.0005
	CD3+CD8+	0.39 (0.16–0.45)	0.35 (0.28–0.68)	0.61 (0.42–0.73)	ns	0.021	ns
	CD4+CD25+	0.02 (0.01–0.04)	0.05 (0.03–0.08)	0.21 (0.16–0.24)	ns	< 0.0001	< 0.0001
	CD4+CD25++CD127^−/low^	0.03 (0.01–0.04)	0.05 (0.03–0.07)	0.05 (0.05–0.07)	0.04	0.001	ns
	CD3+CD69+	0.02 (0.01–0.03)	0.05 (0.04–0.07)	0.06 (0.04–0.08)	0.028	0.007	ns
	CD4+CD69+	0.02 (0.01–0.03)	0.01 (0.01–0.04)	0.05 (0.04–0.08)	ns	0.027	0.011
	CD8+CD69+	0.01 (0.01–0.014)	0.02 (0.01–0.04)	0.09 (0.07–0.12)	0.039	< 0.0001	0.02
	CD3+HLA-DR+	0.11 (0.05–0.18)	0.20 (0.14–0.29)	0.14 (0.08–0.19)	0.011	ns	0.032
	CD4+HLA-DR+	0.05 (0.03–0.11)	0.04 (0.03–0.08)	0.05 (0.04–0.08)	ns	ns	ns
	CD8+HLA-DR+	0.03 (0.02–0.05)	0.05 (0.02–0.09)	0.08 (0.04–0.16)	ns	0.018	ns

*Results are expressed as median (25^th^-75^th^ percentiles). ns represent no statistically significant difference*.

### Lymphocyte Populations in DOBV and in PUUV Infected Patients

**Figure 4:**
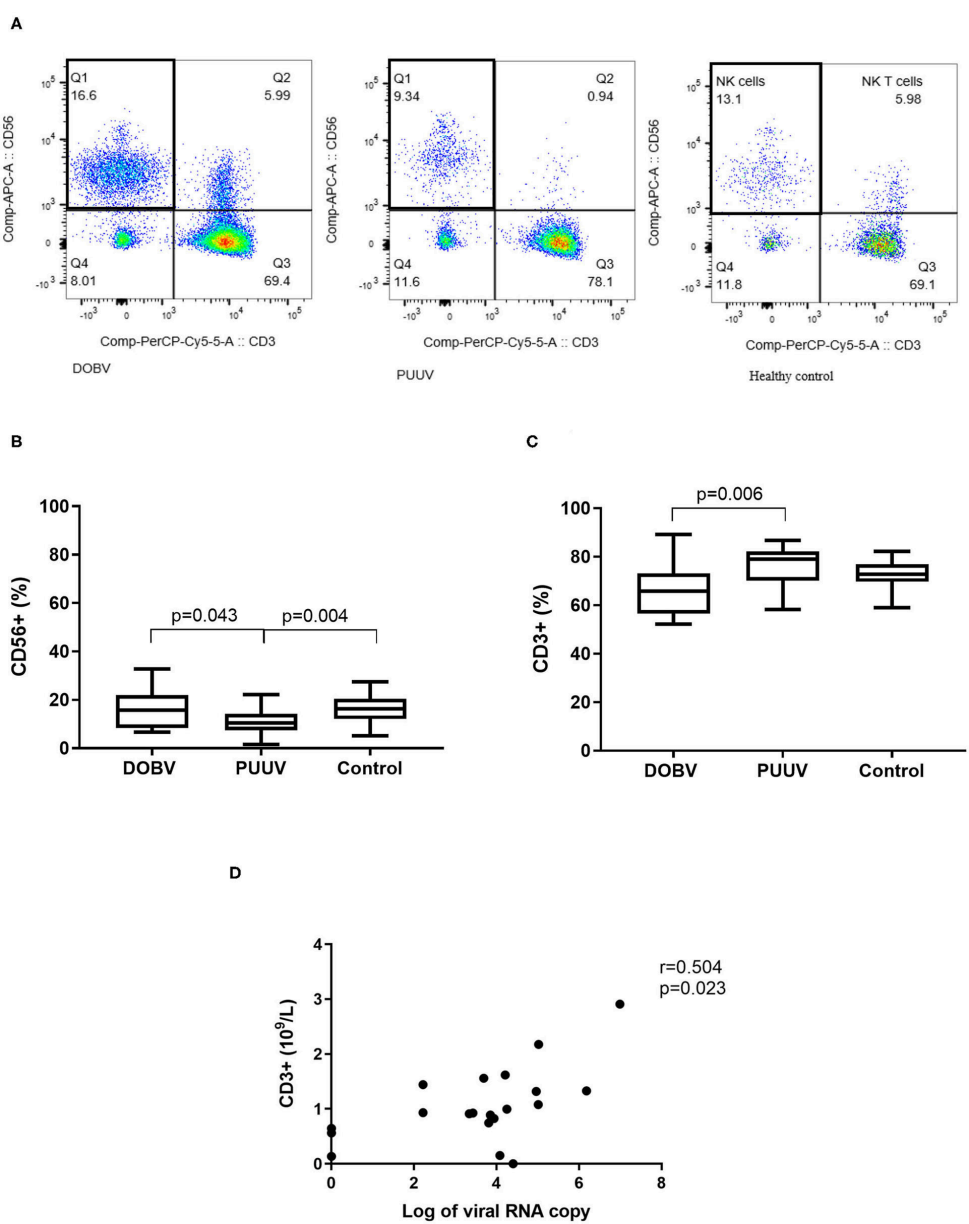
Lymphocyte populations in DOBV and in PUUV infected patients. **(A)** A representative example of CD3-CD56+ NK cells in healthy control and in DOBV and PUUV infected patients. **(B)** Comparison of percentage of NK cells CD56+ between DOBV and PUUV infected patients and healthy control. **(C)** Comparison of percentage of T cells between DOBV and PUUV infected patients and healthy control. **(D)** Correlation between concentration of T cells and viral RNA load in PUUV infected patients (*n* = 20). The non-parametric Kruskal–Wallis test, followed by Dunn's post analysis was used for statistical analyses. The non-parametric Spearman correlation test with 95% confidence interval was performed to compute correlations. A *p*-values below 0.05 were considered statistically significant.

To determine the difference in PBMC profile between DOBV and PUUV infected patients the T cells and NK cells were analyzed. Results indicated that PUUV infected patients had a lower percentage of NK cells (Figure 4A) and a higher percentage of T cells than patients infected with DOBV (Table 2). Percentage of NK cells was 1.6-times lower in PUUV infected patients than in DOBV infected patients (*p* = 0.043) and also 1.5-times lower than in the healthy control (*p* = 0.004), while percentage of NK cells in DOBV infected patients was similar to the healthy control (Figure 4B). On the contrary, percentage of T cells in PUUV infected patients was 1.2-times higher than in DOBV infected patients (p = 0.006). However, percentage of T cells of both PUUV and DOBV infected patients was in the range of the healthy control (Figure 4C). Based on previously observed decreased concentration of lymphocytes in DOBV and in PUUV infected patients, results indicated a lower concentration of T cells and NK cells in both groups of patients in comparison to the healthy control (Table 2). Additionally, concentration of T cells in PUUV infected patients correlated to the viral RNA load (*r* = 0.504, *p* = 0.023; Figure 4D).

### T Helper Cells in DOBV and in PUUV Infected Patients

**Figure 5:**
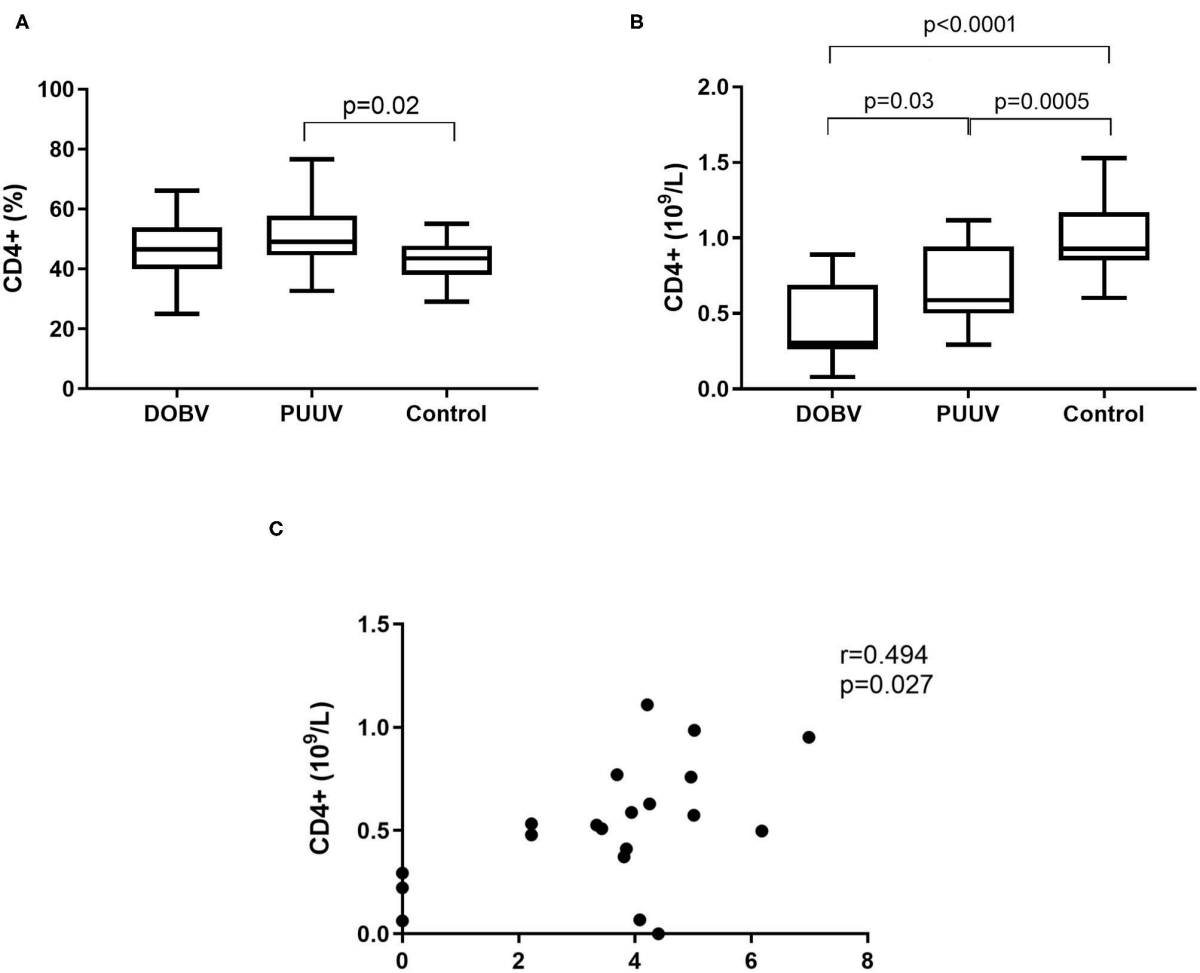
T helper cells in HFRS patients. **(A)** Comparison of percentage of T helper cells between DOBV and PUUV infected patients and healthy control. **(B)** Comparison of concentration of T helper cells between DOBV and PUUV infected patients and healthy control. **(C)** Correlation between concentration of CD4+ cells and viral RNA load in PUUV infected patients (*n* = 20). The non-parametric Kruskal–Wallis test, followed by Dunn's pot analysis was used for statistically analyses. The non-parametric Spearman correlation test with 95% confidence interval was performed to compute correlations. A *p*-values below 0.05 were considered statistically significant.

Further, T helper cells in patients infected with DOBV or PUUV were analyzed. CD4+ cells were significantly elevated in PUUV infected patients (*p* = 0.02; Figure 5A). Concentration of CD4+ cells was 1.9-times higher in PUUV infected patients than in patients infected with DOBV (*p* = 0.03; Figure 5B, Table 2). Due to the observed lymphocyte reduction, the concentration of CD4+ cells was below normal level in both groups of patients (*p* < 0.0001 for DOBV and *p* = 0.0005 for PUUV). Concentration of CD4+ cells in PUUV infected patients correlated to the viral RNA load (*r* = 0.494, *p* = 0.027; Figure 5C).

**Figure 6:**
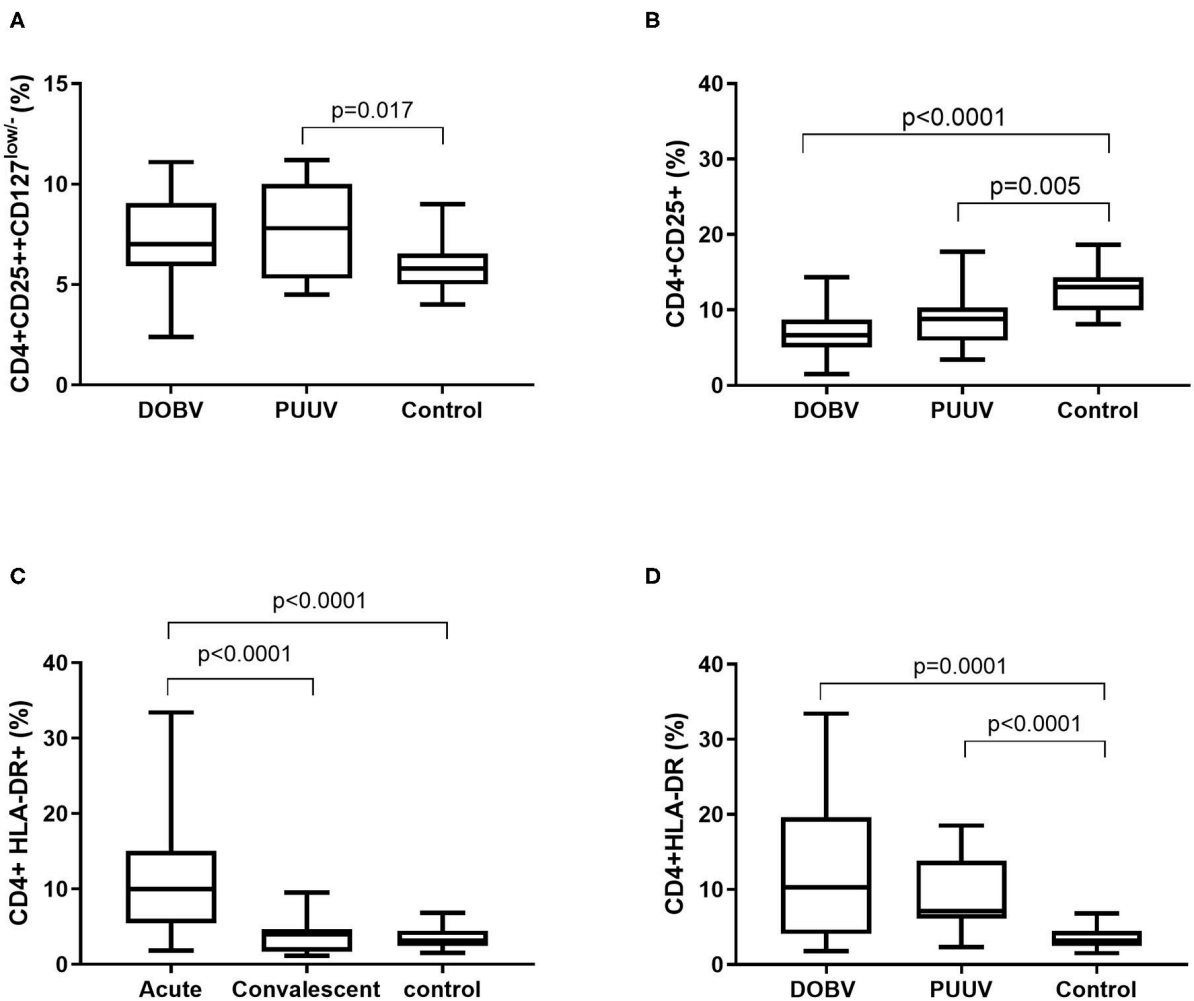
Activation of T helper cells in HFRS patients. **(A)** Comparison of percentage of regulatory T cells between DOBV and PUUV infected patients and healthy control. **(B)** Comparison of percentage of activated T helper cells, which expressed IL-2 receptor between DOBV and PUUV infected patients and healthy control. **(C)** Comparison of percentage of activated T helper cells expressing marker HLA-DR between acute and convalescent samples of HFRS patients and healthy control. **(D)** Comparison of percentage of activated T helper cells expressing marker HLA-DR between DOBV and PUUV infected patients and healthy control. The non-parametric Kruskal–Wallis test, followed by Dunn's pot analysis was used for statistically analyses. A *p*-values below 0.05 were considered statistically significant.

To examine the activation of T helper cells the marker CD25 was analyzed. Activated T helper cells as CD4^+^CD25^+^ and regulatory T cells (T_reg_) as CD4^+^CD25^++^CD127^low/−^ were detected. T_reg_ cells were elevated in PUUV infected patients (Figure 6A). Percentage of T_reg_ cells was 1.3-times higher in PUUV infected patients in comparison to the healthy control (*p* = 0.017; Figure 6A). Similarly, when concentrations were compared, PUUV infected patients had 1.7-times higher concentration of T_reg_ than DOBV infected patients (*p* = 0.04; Table 2). However, when we investigated activated T helper cells which expressed IL-2 receptor (CD4+CD25+) we observed a significantly lower percentages in HFRS patients in comparison to the healthy control (*p* < 0.0001 for DOBV and *p* = 0.005 for PUUV) (Figure 6B). Furthermore, the soluble IL-2 receptor (sIL-2R) in acute serum samples of 11 DOBV and 20 PUUV infected patients was measured and results of chemiluminescent test revealed highly elevated concentration of sIL-2R in both groups of patients. Median concentration of sIL-2R was 2270 u/ml in DOBV infected patients and 2781 U/ml in PUUV infected patients (normal value 623 U/ml).

Additionally, expression of marker HLA-DR on CD4+ cells was analyzed. Results indicated a high activation of T helper cells expressing HLA-DR in acute HFRS. Percentage of activated CD4+ cells was 3-times higher in acute HFRS samples compared to the control (*p* < 0.0001) and 2.5-times higher compared to the convalescent samples (*p* < 0.0001; Figure 6C). Moreover, percentage of activated T helper cells was elevated in DOBV and PUUV infected patients. Percentage of CD4+HLA-DR+ cells was 3-times higher in DOBV infected patients (*p* = 0.0001) and 2-times higher in PUUV infected patients (*p* < 0.0001) in comparison to the healthy control (Figure 6D). However, no significant difference between viruses was detected.

**Figure 7:**
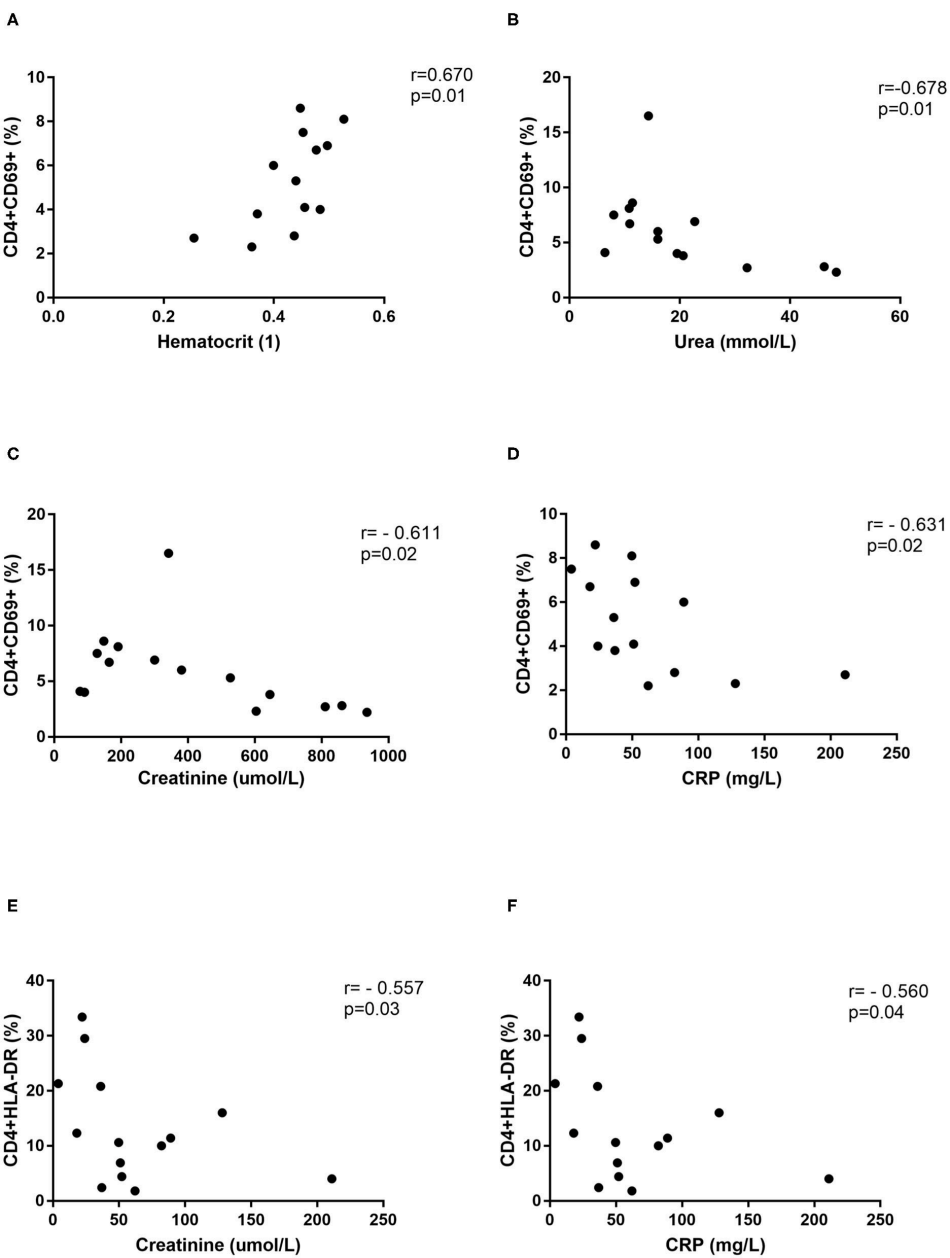
Correlations with clinical parameters. Correlation between hematocrit (*n* = 13) **(A)**, urea (*n* = 14) **(B)**, creatinine (*n* = 15) **(C)**, and CRP (*n* = 14) **(D)** and percentage of CD4+CD69+ cells in DOBV infected patients. Correlation between creatinine (*n* = 15) **(E)** and CRP (*n* = 14) **(F)** and percentage of CD4+HLA-DR+ cells in DOBV infected patients. The non-parametric Spearman correlation test with 95% confidence interval was performed to compute correlations. A *p*-values below 0.05 were considered statistically significant.

Further, correlations with clinical parameters were calculated and results revealed that the activated T helper cells expressing markers CD69 or HLA-DR correlate with the clinical parameters in DOBV infected patients, but not in PUUV infected patients. Percentage of CD4+CD69+ cells positively correlate with hematocrit (*r* = 0.6703, *p* = 0.0147; Figure 7A) and negatively with urea (*r* = −0.6777, *p* = 0.0091; Figure 7B), creatinine (*r* = −0.6107, *p* = 0.0178; Figure 7C) and CRP (*r* = −0.6308, *p* = 0.0181; Figure 7D). Percentage of CD4+HLA-DR+ cells negatively correlate with creatinine (*r* = −0.5571, *p* = 0.0335; Figure 7E) and CRP (*r* = −0.5604, *p* = 0.04; Figure 7F).

### Cytotoxic T Cells in DOBV and in PUUV Infected Patients

**Figure 8:**
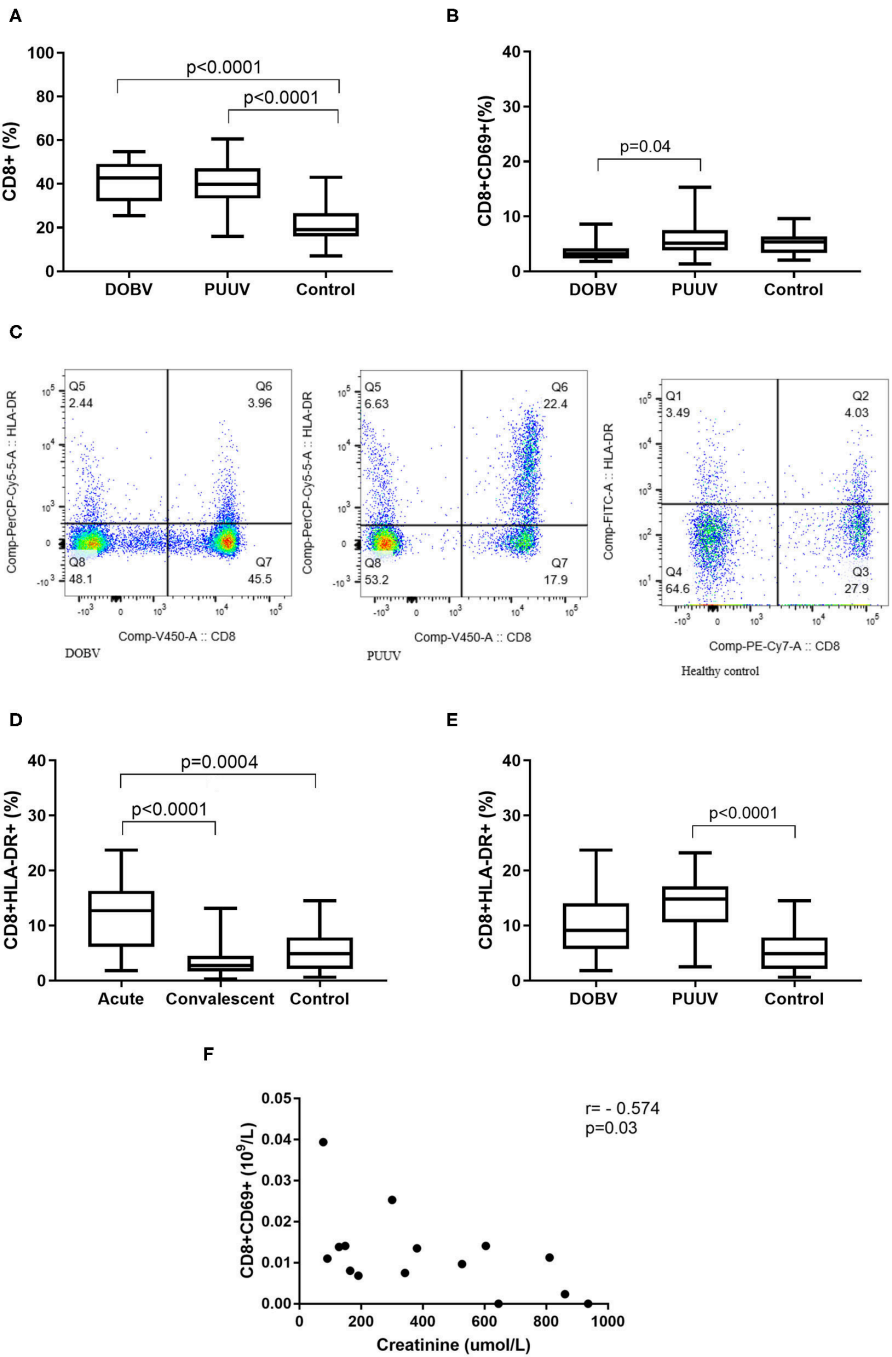
Cytotoxic T cells in HFRS patients. **(A)** Comparison of percentage of cytotoxic T cells between DOBV and PUUV infected patients and healthy control. **(B)** Comparison of percentage of activated cytotoxic T cells, which expressed marker CD69 between DOBV and PUUV infected patients and healthy control. **(C)** A representative example of activated T cells and activated cytotoxic T cells expressing HLA-DR in healthy control and in DOBV and PUUV infected patient. **(D)** Comparison of percentage of activated cytotoxic T cells, which expressed marker HLA-DR between acute and convalescent samples of HFRS patients and healthy control. **(E)** Comparison of percentage of activated cytotoxic T cells, which expressed marker HLA-DR between DOBV and PUUV infected patients and healthy control. **(F)** Correlation between creatinine and concentration of CD8+CD69+ cells in DOBV infected patients. The non-parametric Kruskal–Wallis test, followed by Dunn's pot analysis was used for statistically analyses. The non-parametric Spearman correlation test with 95% confidence interval was performed to compute correlations. A *p*-values below 0.05 were considered statistically significant.

Next, cytotoxic T cells in patients infected with DOBV or PUUV were analyzed. Results indicated significantly elevated percentage of CD8+ cells in DOBV and in PUUV infected patients (*p* < 0.0001; Figure 8A). To examine the early activation of CD8+ cells in HFRS patients, the marker CD69 was analyzed. Percentage of activated CD8+ cells, which expressed marker CD69, was 2-times higher in PUUV infected patients in comparison to the patients infected with DOBV (*p* = 0.04) and the difference was significant (Figure 8B). However, percentage of CD8+CD69+ cells in DOBV and in PUUV infected patients was not elevated according to the healthy control (Figure 8B).

Additionally, expression of activation marker HLA-DR on cytotoxic T cells was investigated (Figure 8C). Similarly to the helper T cells, cytotoxic T cells were also activated in the acute stage of HFRS and normalized in the convalescent. Percentage of CD8+HLA-DR+ cells was 3-times higher in the acute HFRS samples compared to the control (p = 0.0004) and 4.5-times higher compared to the convalescent samples (*p* < 0.0001; Figure 8D). Percentage of activated cytotoxic T cells was significantly elevated only in acute samples of PUUV infected patients and was 3-times higher in comparison to the healthy control (*p* < 0.0001; Figure 8E).

Similarly, activated cytotoxic T cells also correlate with the clinical parameter only in DOBV infected patients. Results revealed that the concentration of CD8+CD69+ negatively correlates with creatinine (*r* = −0.5737, *p* = 0.027; Figure 8F).

## Discussion

Hantavirus pathogenesis in humans results from an interaction of the hantaviruses with the immune system. During a hantavirus infection, viral antigens and different cytokines activate resting lymphocytes, that result in changes of the cell surface phenotype. Although HFRS pathogenesis is not fully discovered, it seems that cytotoxic response of NK cells and T cells has an important role in disease severity. In our study, we investigated different lymphocyte subpopulations between patients infected with DOBV and PUUV.

The immune response has a main role in HFRS manifestation. Mononuclear phagocytes are responsible for the initiation and the regulation of virus specific immune responses. Our results from *in vitro* stimulation of PBMCs are consistent with previously known findings and showed that DOBV and PUUV infect monocytes and activate T helper cells (Temonen et al., 1993; Scholz et al., 2017). During the acute illness, leukocytes are extravasated from blood toward the site of infection. We showed decreased concentrations of lymphocytes and lymphocyte subpopulations in peripheral blood during acute stage of HFRS. Decreased concentrations were especially evident in DOBV infected patients, whereas in PUUV infected patients they correlated with the viral RNA load. The lower lymphocyte concentrations might be observed due to extravasation of the cells from the blood to the tissue, which is compatible with previous studies (Linderholm et al., 1993; Rasmuson et al., 2011). Moreover, absolute monocyte numbers normalized at convalescence, when virus was cleared from the blood (Scholz et al., 2017).

NK cells are an important part of innate immunity against different virus infections (Lodoen and Lanier, 2006; Jost and Altfeld, 2013). Under pathogenic conditions, they rapidly expand and their numbers remain elevated for up to 60 days. In PUUV infected patients a high activation and proliferation of NK cells were already described (Bjorkstrom et al., 2011; Braun et al., 2014). In our study, lymphocyte subtypes were higher activate in PUUV infected patients than in DOBV infected patients. A total CD56+ NK cells were the only lymphocyte subtype, where detected percentage of cells was significantly lower in PUUV infected patients. Studies indicated that lower NK cell numbers in the acute phase of infection could be a result of extravasation cells into tissues (Bjorkstrom et al., 2011). Involvement of NK cells in HFRS severity should be investigated further, however in our study the lowest percentage of total CD56+ NK cells was shown in PUUV infected patients with the mild disease (data not shown).

Also, according to our results, it is likely that a higher T-cell response is associated with a milder HFRS, since a higher percentage of activated T cells was observed in PUUV infected patients. Higher immune response of activated T cells in patients infected with PUUV could contribute to a more efficient virus clearance from the body, as our results showed a lower viral RNA load in patients infected with PUUV in comparison to DOBV infected patients. Also, the correlation between viral RNA load and T cells in PUUV infected patients was observed. The fact that the concentration of activated T cells (CD69+, HLA-DR+), especially activated cytotoxic T cells, are lower in DOBV infected patients, also supports the hypothesis. Decreased immune activation in DOBV infection patients was observed in our previous study, where results showed that DOBV inhibits the IFN type 1-induced antiviral state in PBMCs (Resman Rus et al., 2018). However, important part of immune response is also the effector function of T cells, such as cytokine production, help with antibody production and cytotoxic effects and these factors might be interesting to examine in future studies. Especially as it was shown that, differences between DOBV and PUUV infections in viral load, antibody and cytokine response kinetics could be reflected in differing disease severities and clinical outcome (Korva et al., 2013).

In our study activation markers CD69, CD25, and HLA-DR were investigated on T cells. Our results revealed a higher concentration of activated T cells subsets in PUUV infected patient, which correlated with a milder HFRS in comparison to DOBV infection. The CD69 antigen has been identified as the early activation marker, which is involved in lymphocytes proliferation. It can be a useful early marker of antigen specific activation on lymphocytes *in vitro* (Werfel et al., 1997). Virus-specific cytotoxic T cells are the major effector cells in cellular immune response against a microbial infection and have also an important role in eliminating hantaviruses (Araki et al., 2003), however they may play different roles in different phases of HFRS (Xie et al., 2013). It was shown that SNV-specific CD8^+^ T cells contribute to a HPS disease outcome (Kilpatrick et al., 2004). On the contrary, the study on HFRS patients in China pointed to the primarily protective role of cytotoxic T cells in HTNV infections (Wang et al., 2009). In our study, PUUV infected patients had a significantly higher concentration of activated T cells, especially cytotoxic T cells, expressing CD69 antigen in comparison to DOBV infected patients, which had a significantly lower concentration of activated T cells than the healthy control. This indicates that a protective role of cytotoxic T cells is crucial for a beneficial HFRS outcome (Temonen et al., 1996; Wang et al., 2009; Saksida et al., 2011).

Similarly to CD69 marker, PUUV infected patients also had a higher concentration of T cells that expressed the late activation marker HLA-DR. Up-regulation of HLA-DR suggests better antigen presentation and higher progress toward specific and adaptive phases of the immune response after an infection with PUUV in comparison to DOBV infection, which possibly results in a milder HFRS outcome. Interesting, besides CD8+ cells increased activation was found also on CD4+ cells. The activation of cytotoxic lymphocytes was expected as an effector response to the virus, while the activation of the helper T cells is likely to be due to a high cytokine presence. Our previous study showed that cytokines have an important role in severity of HFRS and only a controlled release of cytokines contributes to a milder disease (Korva et al., 2019). Additionally, activated CD4+ cells correlate with the clinical parameters hematocrit, urea, creatinine, and CRP in DOBV infected patients. Results indicated that the higher activation of helper T cells could contribute to a better renal function, especially in DOBV infected patients with usually more severely impaired renal function.

For a beneficial disease outcome, a balance between regulatory and effector function of T cells is necessary, however insufficient suppression of proinflammatory and effector T-cell activity by inadequate T_reg_ cells response could also contribute to the HFRS pathogenesis (Zhu et al., 2009; Lindgren et al., 2011; Terajima and Ennis, 2011; Rasmuson et al., 2016). However, only a few studies investigated T_reg_ cells in hantavirus infections and those showed controversial results. While HTNV infected patients in China had a significantly lower T_reg_ cells in the acute stage of disease than the healthy controls, PUUV infected patients in Finland had activated T_reg_ cells in the acute disease stage (Zhu et al., 2009; Koivula et al., 2014). In our study, PUUV infected patients showed an elevated percentage of T_reg_ cells (CD4^+^CD25^+^CD127^low/−^) compared to the healthy control and a higher concentration of T_reg_ cells than DOBV infected patients. On the contrary, the activation of effector T cells (CD25+CD4+) was observed by elevated concentrations of serum sIL-2R in DOBV and PUUV infected patients. Higher regulatory function of T cells after PUUV infection could achieve balanced immune responses that clear pathogens without causing excessive immunopathology and probably could contribute to the beneficial HFRS outcome.

In HFRS, the male-to-female ratio for hantavirus disease varies from 2 to 5:1. Our study group consisted of mostly male patients, however the gender ratio between DOBV and PUUV infected patients was similar. Since the gender could play an important role in HFRS immunopathogenesis, further studies of gender influence to HFRS severity are necessary, especially in female DOBV infected patients.

In our study, the difference in PBMCs profile between patients infected with DOBV and PUUV was investigated. Two different roles of activated lymphocyte subpopulations could be involved in HFRS pathogenesis. The higher activation of immune response possibly results in a milder HFRS. This is supported by a higher concentration of different subtypes of T lymphocytes and phenotypic changes, especially the early and late T cell activation markers, in PUUV infected patients. But, it is also possible that DOBV infected patients have a more active immune response in comparison to PUUV infected patients, but because of extravasation of cells into infected tissues this could not be detected in peripheral blood. A more severe course of the disease and hemorrhagic manifestations in DOBV infected patients support this hypothesis. However, more studies on local immune responses are necessary to confirm the direct involvement of T lymphocyte subsets on the HFRS immunopathology.

## Data Availability Statement

The raw data supporting the conclusions of this article will be made available by the authors, without undue reservation.

## Ethics Statement

The study was approved by the Republic of Slovenia National Medical Ethics Committee (69/03/12 and 30/04/15). All participants gave an oral and a written informed consent. The study was conducted according to the principles expressed in the Declaration of Helsinki. The patients/participants provided their written informed consent to participate in this study.

## Author Contributions

TAŽ, AI, KR, and AK: conceived and designed the experiments. KR and AK: performed the experiments and analyzed the data. KR, AK, MK, AI, MP, and TAŽ: wrote the paper. All authors contributed to the article and approved the submitted version.

## Conflict of Interest

The authors declare that the research was conducted in the absence of any commercial or financial relationships that could be construed as a potential conflict of interest.
